# Cosmetic ethnobotany practiced by tribal women of Kashmir Himalayas

**Published:** 2014

**Authors:** Hamayun Shaheen, Jaweria Nazir, Syeda Sadiqa Firdous, Abd-Ur-Rehman Khalid

**Affiliations:** 1 *Department of Botany, University of Azad Jammu & Kashmir Muzaffarabad, Pakistan*; 2 *Department of Plant Pathology, the University of Poonch Rawalakot, Azad Jammu & Kashmir, Pakistan*

**Keywords:** *Cosmetic herbs*, *Himalayas*, *Skin treatment*, *Tribal women* ‎

## Abstract

**Objective:** Himalayan mountain populations have been dependent upon indigenous plant resources for their health care for many years. Tribal women are interested in use of local herbs for cosmetic purposes. The present work is based on the results of research conducted on cosmetic uses of some important plants by the tribal women in District Poonch, Azad Kashmir Pakistan.

**Materials and Methods:** An ethno botanical survey was carried out during summer 2012. The data were collected from 310 female informants from 16 villages using questionnaire method and semi structured interviews.

**Results:** A total of 39 plants species belonging to 20 families, being used for various cosmetic purposes were recorded. Indigenous species are traditionally used by the locals for problems including acne (16%), hair growth (11%), bad breath (12%), facial spots (9%), allergy, (9%), fairness (8%), wrinkles (8%), eye and lip care (9%). Seventy different recipes were recorded to be practiced by locals using herbal parts. The major plant parts utilized in herbal recipes included fruit (32.8%), Leaves (25.2%), seeds (13.4%) and roots (8.9%). Women of older (>30 years) age group showed greater (67%) response regarding knowledge and practice of cosmetic herbs.

**Conclusion:** This study was the 1^st^ ever project focusing on cosmetic perspectives of ethno-botany in the area. Our study contributes to an improved understanding of ignored aspect of cosmetic ethnobotany among the local women. Further detailed investigations are recommended to record and preserve precious ethno-botanical knowledge of the area.

## Introduction

Human civilizations have developed and relied upon domestication of plant species for forage, medicinal uses, fiber culinary and cosmetic purposes (Kala, 2007 ▶). Ethnobotanists aim to document, describe and explain complex relationships between cultures and plants, focusing primarily on how plants are used, managed and perceived across human societies (Acharya et al., 2008 ▶). The field of ethnobotany requires taxonomic, morphological, ecological and anthropological skills to understand the cultural concepts around the perception of plants (Ali and Qaisar, 2009 ▶; Everest and Ozturk, 2005 ▶). Medicinal plants are globally used to treat a wide range of ailments, infections and disorders. It is estimated that about 70% of Himalayan population is dependent upon ethnomedicine for their primary health care (Shaheen et al., 2012a ▶). The ethnobotanical practices are very popular among the locals due to ease of availability, good results as well as minimum side effects. More than 10% (>600/ 5700) plant species of Pakistani flora are reported to have medicinal importance (Shinwari, 2002 ▶; Ajaib et al., 2010 ▶). 

Herbal recipes had been used by women through years for enhancement and preservation of beauty (Khan and Khatoon, 2007 ▶). Apart from traditional ethno-cosmetic applications of local herbs, efforts are in progress to formulate and develop personal care products based on these natural resources, termed as herbal cosmetics (Afzal et al., 2009 ▶). There is an increasing demand of these herbal cosmetics due to their natural purity, little or no side effects and impressive results (Hamayun et al., 2006 ▶; Khan et al., 2007 ▶). Scientific evidence supports the fact that phytochemicals are very effective in smoothening, calming, restoring and healing of skin and hair; as well as perfuming and correcting body odors (Ghimire et al., 2006 ▶).

The tribal women population of Kashmir Himalayas is very laborious and dynamic; and by instinct conscious about cosmetic applications of local herbs (Shinwari et al., 2000 ▶). In male dominated, conservative religious mountain tribes, women are reluctant, discouraged and shy to discuss their cosmetic problems with doctors or family member (Shinwari et al., 2006 ▶; Dar, 2003 ▶). A tragedy of the modern time is that the precious cosmetic ethnobotanical knowledge is disappearing quickly. Due to the lack of interest and knowledge the younger generation prefers allopathic medicines and cosmetic products (Uninal et al., 2006 ▶). Preservation of the values of plants can only be maintained with the help of the indigenous people who have used this knowledge for centuries (Shrestha and Dhillion, 2003 ▶). Although researchers have conducted a lot of work in the field of ethnobotany, yet its cosmetic aspect has never been focused in this area previously. The main objective of this research was to explore the cosmetic value of plants and make the new generation aware about it. 

## Materials and methods

Azad Kashmir forms the lower hills of Himalaya and covers an area of 13, 297 km^2^. Total population of Azad Kashmir is estimated at around 4.5 million with a Population density of 343.5/km^2^. The area of Hajira lies in District Poonch, located at latitude 33°46'16.58"N, longitude 73° 53'46.67"E and an elevation of 1545m. The topography is hilly and mountainous with valleys and plains at some places. The total rainfall is about 149.93mm/year. Floristically the study area is located in western Himalayan moist temperate province (Shaheen et al., 2012 a ▶, b ▶). 

Field studies were carried out during summer and spring 2012. About 320 women from 16 villages (20/village) were interviewed. Informants were classified in different age groups. One hundred and ten informants were below the age of 30; hundred were in the age group of 30-50 years; and 110 were above the age of 50. Ethno botanical and demographic information was gathered from the respondents by using semi structured, open ended questionnaire. The questionnaire focused on informan’s knowledge of the cosmetic herbs, collection, uses and recipe preparation; major infections and disease treated. Local names, distribution of plants, dose preparation, medium of intake and application of cosmetic recipe were also asked from the informants. The data were arranged according to taxonomic identification of plants, their uses and local names. The data were further analyzed for basic categorization of the respondents’ age, literacy, gender, use preferences, parts of medicinal plants used, recipes preparation and mode of administration. 

## Results

This study provides information on the indigenous uses of 39 important plants belonging to 20 families by the local women for various cosmetic purposes (Table 1, 2). 

**Table 1:** Cosmetic ethnobotanical applications recorded from the study area

***Species***	**Local Name**	**Part used**	**Application**	**Recipe **
***Allium sativum ***	Thom	cloves	Toothache	Garlic extract is made and cotton is dipped in it. This cotton is applied on teeth. Extra water is released from gums and pain gets reduced.
			pimples	Garlic cloves are crushed and powder is applied over the pimples. It is than washed off with cold water and dried thoroughly.
			Nails	Garlic slices are rubbed on nails. This makes nails stronger.
***Aloe barbedansis ***	Kanwar gandal	Leaves	Acne	Aloe leaf gel is pricked out in a glass and this extract is drunk daily for some days.
***Allium cepa ***	Pyaz	bulb	Hair growth	Outer peels of onion are blended and sieved to extract the liquid. This extract is applied on scalp.
			exfoliate dead skin	Flour, onion juice and milk are mixed to form a thick paste. This paste is applied on face and neck for 15 – 20 minutes until it dries.
			facial massage	Fresh onion juice is mixed with fresh yogurt. It is then massaged on entire face in gentle circular movement for 15 minutes every day.
***Adhatoda vesica***	Baykar	Leaves	skin rots	Leaves are put into the Luke warm water and left for few minutes. Hands are dipped in this water.
***Artemisia rotifolia ***	Afsanteen	Leaves, scales	Hand boils	Dried leaves and stem scales are chipped into a fine powder. It is then used with water for hand boils.
***Berberis lyceum***	Sumbalo	Bark	Pimples	Bark is chopped into a fine powder. This powder is used daily for the removal of pimples.
***Curcuma picta ***	kachoor	Roots	Pimples	Root powder is used daily with water to reduce pimples.
***Cucumis sativus***	kheera	Fruit	dark circles	1: Cucumber slices are placed on eyes for 5 to 10 minutes. 2: Cotton is dipped in cucumber juice. This cotton is applied on eyelids for 10 to 20 minutes for fairness.
***Citrus Limon ***	Limo	Fruit	tooth tartar	Dry lemon peels are Grinded up and some quantity of salt is added. This mixture is used daily with brush to remove the tartar of tooth.
			Wrinkles	Lemon extract is applied on face wrinkles twice or thrice a day.
			nails care	1: Lemon juice is mixed with Luke warm water. A ball of cotton is dipped in this mixture and applied on the nails. This removes the nails dust and gives shiny appearance to nails. 2: Lemon is cut and rubbed on nails for the shining of nails.
			Skin cracks	Lemon juice is mixed with glycerin to form a mixture. This mixture is applied on hands before sleeping.
			Dandruff	Lemon juice and coconut oil are mixed to get a paste. This paste is applied and massaged on hair.
			Hair shine	A mixture of lemon juice along with mustard oil is made. This is applied and massaged on hair and then washed.
			Skin softness	Lemon juice is mixed with almond oil, egg yolk and stirred until a fluffy appearance. This mixture is applied on the skin for 10 minutes and then washed with Luke warm water.
***Citrullus vulgaris***	Tarbooz	Seeds	lip cracks	Unripen seeds are crushed and mixed with water; applied daily on lips at night and washed with Luke warm water next morning.
***Coriandrum sativum***	Dhaniya	Leaves	mouth smell	Leaves are chewed for few minutes.
***Curcuma longa***	Haldy	Roots	Wrinkles	Turmeric powder with milk is mixed up very gently to make a paste and applied daily at face wrinkles.
			facial hair	Turmeric is mixed with chick pea flour and milk to from a thick paste. It is applied on face daily on night for 10-15 minutes and then washed with Luke warm water.
***Citrus reticulate***	Malta	Fruit	Skin and nail cracks	Orange juice is mixed gently with honey and applied on hands for skin cracks as well as shining of nails.
***Daucus carota***	Gajar	Root	Fairness	Carrot juice is extracted and cotton is dipped in it. It is daily massaged on face.
***Dioscoria deloidea ***	Kala ganda	Root	Pimples	Dry roots slices are blended to form a fine powder. This powder is used daily with water.
***Echniopsis mamilosa ***	Thohar	Stem	Dandruff	Herbaceous stem juice is extracted. It is used daily for few days to remove the extra dryness of skin and dead skin layer of dandruff.
***Foeniculum vulgare***	Sounf	Seeds	mouth smell	1: Funnel seeds are chewed twice or thrice a day. 2: Funnel seeds are boiled in water. This water is used twice a day.
***Ficus palmate***	Phagwara	Fruit	Freckles	Figs are blended with yogurt to form a thick paste. This mixture is massaged all over the face and neck in a gentle circular movement for 10-15minutes. Than it is washed with Luke warm water.
			viral warts	The fig milk is applied on viral warts present on skin.
***Jasminum officinale***	Chambayli	Leaves	Mouth freshness	The leaves are boiled in water for half an hour. Mouth is rinsed daily in the morning thoroughly for 1-2 minutes for freshness.
***Juglans regia***	Akhrot	bark, leaves	Tartar	Outer bark and leaves are used as a misvak to remove the tartar of teeth.
***Lycopersicum esculentum***	Tamatar	Fruit	face spots	Fresh tomato extract is applied on skin daily to prevent face spots.
			sun block	After washing the face with Luke warm water tomato extract is applied to face and messaged for 10-15 minutes. This proves to be an effective sun block for face.
			skin glowing	Tomato is mixed with honey. This mixture is applied on face and neck. It is washed after fifteen minutes
			open pores and black heads	Sliced Tomato pieces are rubbed on face and left for fifteen minutes. Tomato has acidic trait and contains vitamin c and K which helps in cleansing. This recovers the open pores and removes black heads.
***Mentha longifolia***	Podina	Leaves	Fairness	Mint leaves are boiled in water for half an hour. This extract is taken at morning before breakfast about 1/3 cups daily.
			Blackheads	Extract of mint leaves is applied on the affected areas of skin. It proves as an excellent skin cleanser and curing blackheads.
			Pimples	Extract of mint leaves mixed with oat is applied on pimples and washed with cold water after twenty minutes.
			mouth smell	Mint leaves are boiled for half an hour. This boiled water is effective in reducing mouth smell.
***Melia azadirachta***	Derek	leaves	Freckles	Fresh leaves are crushed to get an extract. It is taken daily to remove freckles.
		seeds	eyes swelling	Seeds are crushed into a fine powder and applied on eyes to treat swelling of eyes.
***Musa paradisiaca***	Kayla	Fruit	lips blackness	Inner pulp of banana peel is taken and mixed with lemon juice. This is applied on lips for five minutes twice a week.
***Nigella sativa***	Kalongy	Seeds	Freckles, eyes circles.	Seeds of *Nigella sativa* are used daily with honey to cure freckle and eyes dark circles.
***Olea Ferrugenia***	kahu	Seeds, leaves	Dandruff	1: Seeds are crushed in mortar to form a powder. This powder is mixed with the mustard oil and left for few days and then used for dandruff. 2: The leaves of Olea are boiled in water. The hair are washed with this water.
***Pyrus malus***	Saib	Fruit	facial spots	Apple is crushed with turmeric and equal proportion of rose water and lemon juice to form a paste. This paste is applied daily on face for two weeks for curing unwanted spots.
			Wrinkles	Apple is mixed with unsalted butter, honey and egg yolk. This mask provides intense moisturizing and antiaging effects when applied for twenty minutes on the face.
***Prunus persica***	Arhu	Leaves	skin spots	Leaves are crushed and applied on the white circles of face.
***Pyrus pashia***	Batangy	Fruit	eyes dark circles	*Pyrus pashia* is eaten in the dried form to remove the dark circles around the eyes.
***Raphanus sativus***	Mule	Leaves, seeds	facial spots	1: Leaves are crushed and applied on facial skin. 2: Grinded radish seeds and yogurt mixture are applied on the face. This treatment also removes unwanted spots from face.
			Itching	Fresh and healthy Radish is taken, and its epidermis is peeled off. It is cut into small pieces and blended. After blending, juice is sieved and applied on face for three days.
			Acne, blackhead	Radish is cut into two halves longitudinally and hollowed internally. This hollow portion is filled up with normal washing soap and then closed with the second separate portion and left into sun light for eight days. This soap is rubbed on the facial skin until it gets absorbed in skin. Then this soap is applied on blackheads and acne to got remarkable effects.
***Rosa indicia***	Gulab	Rose petals	Fairness	Dry rose petals are grinded up. Chick pea flour and water is added in this ground mixture to make a past. This paste is applied daily on face for fairness.
			Freshness	Rose water is applied on facial skin to prevent loss of extra water from skin and keeping the skin fresh.
			face scars	Rose water is mixed with clay (multani matti) to make a paste. It is applied on face for 15 minutes for one week daily.
***Solanum melongen***	baingan	Fruit	feet smell	Brinjal is cut into pieces and boiled in water for half an hour. The feet are washed with this Luke warm water for a weak.
***Spindus sponaria***	Ranthy	Fruit	hair growth and shine	Dry peels are boiled in water. This water is used for hair wash for promoting hair growth and shine.
***Solanum nigrum***	Kach mach	fruit	inflammation	*Solanum nigram* is eaten daily for few days. It removes inflammation of facial skin due to summer sun light.
***Trachyspermum ammi***	Ajwain	Seeds	facial black spots	1tsp of ajwain powder is mixed with 1cup of curd for two hours. This paste is applied on face, particularly on black spots.
***Vitis vinifera***	Angoor	leaves	face spots	Leaves are crushed; their juice is extracted and taken orally. This treatment kills the nematodes in the abdomen and ultimately removes the white spots from skin.
***Zizipus jujube***	bair	leaves	hair growth	1: The Jujuba leaves are grinded and applied on hair before 40minutes of washing. This induces better hair growth. 2: Jujube leaves are soaked in water for few minutes. Then hair are Washed with this water to promote hair growth.
***Zingiber officinale***	Aderak	Roots	Acne	Ginger juice is applied on acne affected area.
***Zanthoxylum alatum***	Timber	leaves	Itching	Leaves are boiled in water. This water is used for bathing daily to avoid itching.
		Shoot	Tartar	The herbaceous branches are used as a brush (misvak) to treat mouth tartar.

**Figure 1:**
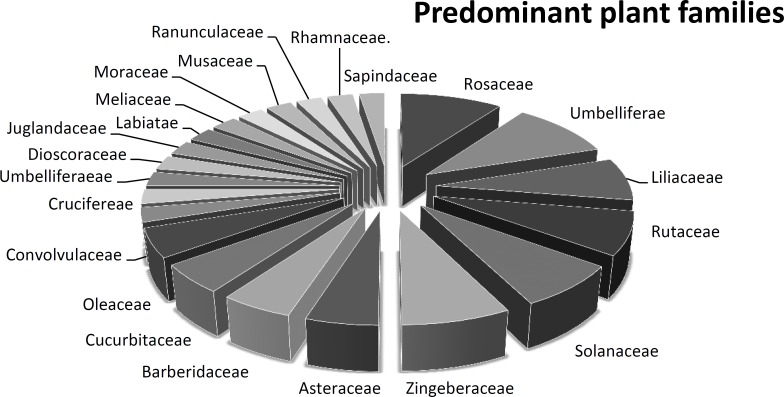
Predominant plant families utilized in cosmetic ethnobotany in the area

Predominant families included Rosaceae and Umbelliferae with 4 members each; Solanaceae, Liliaceae, Zingeberaceae, Rutaceae with three members each. Berberidaceae, Cucurbitaceae and Oleaceae had 2 members each whereas Acanthaceae, Cruciferae, Juglandaceae, Meliaceae, Musaceae, Convoluvlaceae, Moraceae, Ranunculaceae, Rhamnaceae, Asteraceae, Labiateae had 1 member each (Figure 1; Table 2). 

Major plant species having cosmetic applications included *Citrus Limon* with 8 recipes followed by *Lycopersicum esculentum, Mentha longifolia, Raphanus sativus* with 4 uses and *Rosa indica, Allium sativum* and* Allium cepa* with 3 uses each (Table 1).

**Table 2:** List of plants species having cosmetic applications in the area

** No.**	**Botanical name**	**Local name**	**Family**
		*Adhatoda vesica* Miller.	Baykar	Acanthaceae	
		*Allium sativum *Linn.	Thom.	Liliacaeae	
		*Allium cepa *Linn	Pyaz	Liliacaeae	
		*Aloe barbedansis *(L.)Burm	Kanwar gandal	Liliacaeae	
		*Artemisia rotifolia *Spreng.	Afsanteen	Asteraceae	
		*Berberis lycium *L.	Sumbalo	Barberidaceae	
		*Citrullus vulgaris *(Schrad ex Ecki & Zey*h)*	Tarbooz	Cucurbitaceae	
		*Citrus limon *(Linn.)Burm.f	Limo	Rutaceae	
		*Citrus reticulate *Blanco, fl.	Malta	Rutaceae	
		*Coriandrum sativum *Linn.	Dhaniya	Umbelliferaeae	
		*Cucumis sativus *Linn.	Kheera	Cucurbitaceae	
		*Curcuma longa *Linn.	Haldy	Zingirberaceae	
		*Curcuma picta* Roxb.ex.skornick	KacHoor	Zingeberaceae	
		*Daucus carota *Linn.	Gajjar	Umbelliferae	
		*Dioscoria deloidea *GN	Kala ganda	Dioscoraceae	
		*Echniopsis mamilosa *	Dhaniya	Umbelliferaeae	
		*Ficus palmate *L.	Phagwarah	Moraceae	
		*Foeniculum vulgare *Mill.	Sounf	Umbelliferae	
		*Jasminum officinale *Linn.	Chambayli	Oleaceae	
		*Juglans regia *Linn.	Akhrot	Juglandaceae	
		*Lycopersicum esculentum *Mill.	Tamatar	Solanaceae	
		*Melia azadirachta* Linn	Derek	Meliaceae	
		*Mentha longifolia* L.	Podina	Labiatae	
		*Musa Paradisiaca *Linn*. *	Kayla	Musaceae	
		*Nigella sativa *L*. *	Kalongy	Ranunculaceae	
		*Olea ferrugenia *Royle.	Kahu	Oleaceae	
		*Prunus persica* (L.) Stokes	Arhu	Rosaceae	
		*Pyrus malus *L.	Saib	Rosaceae	
		*Pyrus pashia *L.	Batangy	Rosaceae	
		*Raphanus sativus *Linn	Mule	Crucifereae	
		*Rosa indicia *Linn*.*	Gulab	Rosaceae	
		*Solanum melongen*. L.,	Baingan	Solanaceae	
		*Solanum nigrum *Linn	Kach mach	Solanaceae	
		*Spindus sponaria *L.	Ranthy	Sapindaceae	
		*Trachy spermum ammi *(L.)Sprague	Seeds	Umbelliferae	
		*Vitis vinifera *Linn*.*	Angoor	Berberidaceae	
		*Zanthoxylum alatum* Dc	Timber	Rutaceae	
		*Zingiber officinale *L.	Aderak	Zingiberaceae	
		*Zizipus Jujuba *Mill	Bair	Rhamnaceae.	

The local women were using the plants for various cosmetic purposes in 70 different recipes (Table 1). The prominent problems were pimples, acne (16%); Hair growth, shine and dandruff (11%); Mouth tartar and smell (12%); Facial spots (9%); Allergy, itching and warts (9%); Skin freshness and softening (8%); Skin fairness (8%); Wrinkles and freckles (8%); Eyes care (6%); Lips care (3%); body smells (3%); and facial hair (2%). (Table 1, Figure 3). 

The major plant parts utilized in herbal recipes included fruit (32.8%), Leaves (25.2%), seeds (13.4%), roots (8.9%), Bulbs and rhizomes (4.5%), bark (2.9%) and stem (2.9%) (Figure 4)

**Figure 2:**
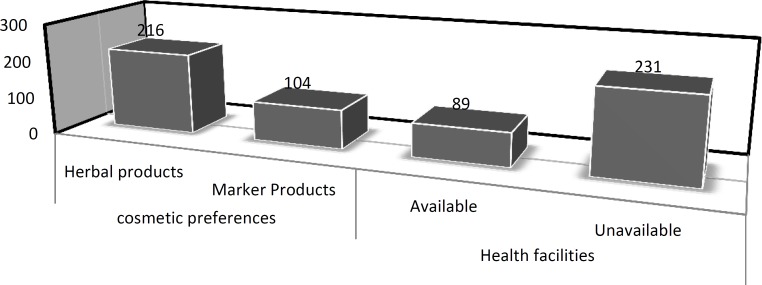
Locals preferences about cosmetics and availability of health facilities

**Figure 3:**
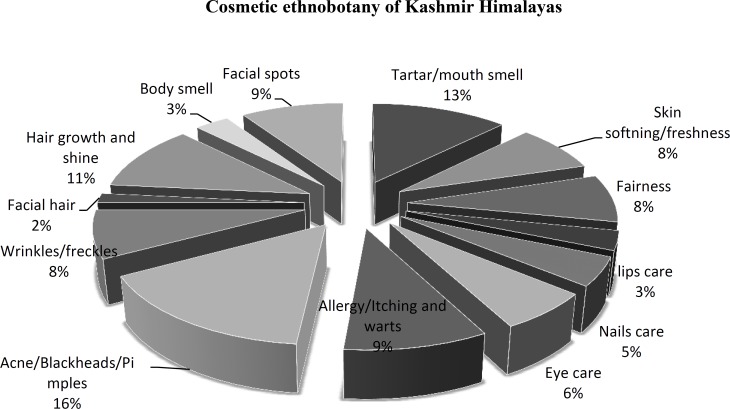
Major applications of cosmetic herbs among the locals in the area

**Figure 4:**
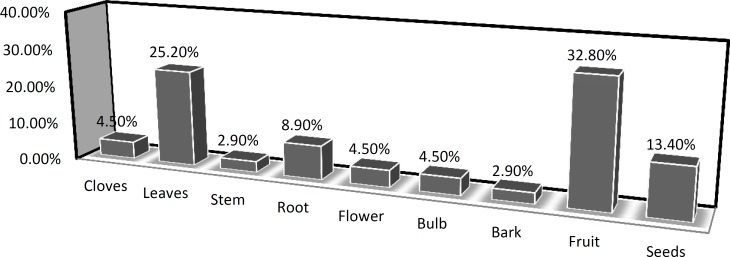
Proportion of herbal parts used in cosmetic recipes

**Table 3:** Age group and literacy level frequencies of the respondents in the area

**Age group**	**Individuals**	**%age**	**Literacy rate **	**Individuals**	**% age**
20-30	110	35.5	Illiterate	183	59
30-50	100	32.3	School	78	25.2
>50	110	35.5	College	39	12.6
			University	10	3.3

## Discussion

The respondents of the questionnaire represented a diverse array of tribal women including literate, illiterate, young and elders. Among the 310 informants, the largest (67.9%) proportion was of elderly, above 30 years of age (Table 3). More than half of the respondents were illiterate (59%). Seventy one percent respondents reported unavailability of health facilities, especially for skin, hair and other cosmetic problems which reflect that herbs are the only available choice for cosmetic purposes in the area (Figure 2). The younger age group showed greater interest in synthetic market cosmetic products as compared to that in elder groups. The maximum response (69%) was observed in the older age group i.e. >30 years (Table 3) indicating popularity of cosmetic ethnobotany in elder tribal women (Khan et al., 2011 ▶; Shah & Joshi, 2009 ▶). These results reflect that indigenous knowledge is well established but seems to be decreasing in the younger generation.

Mode of administration or method of intake was different for different plants. Some plants were used orally or some plants were used externally for the treatment of many skin disorders. The women of the area prefer cosmetic ethno botany because in the remote areas, women have no alternative choices, poverty and they have faith in plants and trust in the effectiveness of folk lore herbal remedies (Qureshi et al., 2009 ▶). These ethnomedicine are natural and beneficial for the health because there are no impurities in this type of medicines which are prepared by people themselves (Ahmed et al., 2009 ▶). Women also prefer ethnomedicine because allopathic medicines are expensive as compared to natural ethno medicine (Bekalo et al., 2009 ▶). 

Unavailability of the modern heath care facilities is another important reason for the tribal women to depend upon the herbal resources as the only available choice. Seventy two percent respondents reported unavailability of modern health facilities regarding cosmetic purposes (Figure 2). Our results revealed that the older generation possessed sufficient knowledge about cosmetic herbs as compared to younger generation. The younger generation seemed to be involved in synthetic cosmetics inspired by intensive media campaigns and advertisements (Kumar et al., 2009 ▶). 

The results of this research showed that women’s information regarding the medicinal plant used, part used, dose preparation and application was highly credible. However our study was the 1^st^ ever effort focusing on cosmetic utilization of local herbs and there is further need of detailed and intensive investigations with particular reference to herbal cosmetics in Kashmir Himalayas. The rapid socio economic and cultural transformations in Himalayas have brought about changes in ecology and people plant interaction (Khan et al., 2012 ▶). The indigenous knowledge about local herbs is declining in several regions (Kassam et al., 2011 ▶). 

The findings of the present research are in harmony with the results of ethnobotanical investigations of Bekalo et al., 2009 ▶ in Ethopia; Everest & Ozturk, 2005 ▶ in Turkey; Kala, 2007 ▶ in Indian Himalayas; Kumar et al., 2007 ▶ in Jammu and Kashmir, India; and **Shaheen et al*.,* 2012 **in Azad Jammu & Kashmir, Pakistan. The results of the present study also indicate similar declining trends about cosmetic herbs in the area, particularly in younger generation. Several other research studies also support this fact that there is an urgent need for preserving indigenous knowledge in Himalayas (Jan et al., 2009 ▶; Kala & Mathur, 2002 ▶; Coopsoomay & Naidoo, 2012 ▶). We suggest that young generation should be trained and made aware about importance, sustainable utilization as well as domestication of the precious cosmetic herbs. 
